# Effects of Dislocation Density Evolution on Mechanical Behavior of OFHC Copper during High-Speed Machining

**DOI:** 10.3390/ma12152348

**Published:** 2019-07-24

**Authors:** Hongguang Liu, Jun Zhang, Xiang Xu, Yutong Qi, Zhechao Liu, Wanhua Zhao

**Affiliations:** 1 School of Mehcanical Engineering, Xi’an Jiaotong University, Xianning West Road 28, Xi’an 710049, China; 2 State Key Laboratory for Manufacturing System Engineering, Xi’an Jiaotong University, Yanxiang Road 99, Xi’an 710054, China

**Keywords:** high-speed machining, dislocation density, mechanical behavior, microstructure

## Abstract

This paper aims at investigating the change in material behavior induced by microstructure evolution during high-speed machining processes. Recently, high-speed machining has attracted quite a lot of interest from researchers due to its high efficiency and surface quality in machining large-scale components. However, the material behavior could change significantly at high-cutting speeds compared to the conventional cutting conditions, including their microstructure and t mechanical response. This is due to the basic physics of material at microscopic levels with high strain, high strain rates, and high temperatures. In this study, the dislocation density-related microstructure evolution process and mechanical behavior of OFHC (Oxygen-free high-conductivity) copper in high-speed machining with speeds ranging from 750 m/min to 3000 m/min are investigated. SEM (Scanning Electron Microscope) and advanced EBSD (Electron Backscattered Diffraction) techniques are used to obtain high-quality images of the microstructures and analyze the dislocation density and grain size evolution with different cutting speeds. Moreover, as material plasticity is induced by the motion of dislocations at micro-scales, a dislocation-density based (DDB) model is applied to predict strain-stress and microstructure information during the cutting process. The distributions of dislocation densities, both statistically stored dislocations (SSDs) and geometrically necessary dislocations (GNDs), are obtained through simulation and experimentation, respectively. The results show that the fluctuation in the cutting forces at high cutting speeds is induced by the specific evolution and distribution of the dislocation density under high strain-rates, and the periodical distribution of sub-surface and fracture behavior during chip separation, which are also found to be influenced by the evolution of the dislocation density.

## 1. Introduction

High-speed machining is becoming more and more popular in manufacturing, such as the large-scale structural components in the aerospace industry, due to its high cutting efficiency, low cutting forces, and high surface quality. The investigation of deformation mechanisms during high-speed cutting processes can provide a better understanding of the basic physics of machining. Ultan and Özel [1] reviewed the microstructure alterations during the surface generation process under machining and concluded that the microstructure evolution is one of the dominant factors influencing the performance of components. Guo et al. [2] investigated the microstructure evolution of OFHC copper during the cutting process and pointed out that grains would be refined to very small nano-scale sizes under the severe plastic deformation (SPD) processes during cutting, which could be attributed to a mixed DRX (dynamic recrystallization) mechanism, and this technique could be used to control the microstructures produced in the machined surface. Pan et al. [3] introduced a method to model the cutting forces of Ti6Al4V alloys with the consideration of microstructure evolution. Moreover, during high-speed machining processes, microstructures evolve differently from the conventional cutting conditions, and this difference of material behavior at the micro-scale can finally influence the mechanical response at the macro-scale. Meanwhile, Yang and Liu [4] presented a method that uses milling to modify the microstructure of machined surfaces and obtain the desired mechanical behaviors for components, and Li et al. [5] pointed out that the mechanical and physical properties of machined parts could be changed due to the microstructure evolution as well. Shyha et al. [6] also found that the microstructure alteration in machined surfaces is influenced by cutting speeds and chip formation processes during milling of the Ti6Al4V alloy, which finally influences the performance of the components. Above all, the microstructure is one of the key factors for obtaining good performance of the final components and has attracted more and more interest from researchers.

During high-speed machining processes, the effects of microstructure evolution on material deformation are more significant from both the macro and micro sides. On the macro side, Sutter and List [7] reported that chip morphology change from continuous to serrated with the increase of cutting speeds and finally turn to fragmented when the cutting speeds are elevated to very high. Meanwhile, cutting forces have a large fluctuation due to the change of microstructure evolution inside the chip area. On the micro side, Duan and Zhang [8] investigated the adiabatic shear banding during the high-speed cutting of steels and found that DRX occurs inside the shear bands, which leads to a change in the mechanical behavior like variation in the cutting forces and the formation of serrated chips. Moreover, Wang et al. [9] also reported a fracture mechanism transition from ductile to brittle at high cutting speeds due to the significant change in the stress state and fracture characteristics induced by microstructure evolution inside the cutting area. Then Wang et al. [10,11] analyzed the microstructure evolution-induced change of micro-hardness in chips and machined surfaces of Ti6Al4V alloys and Inconel 718 and used it to explain the serrated chip formation mechanisms and the change in mechanical behavior during the chip formation process. Childs et al. [12] raised a combined flow stress and failure model to describe the serrated chip formation process of Ti6Al4V alloy within a wide cutting speed range, which revealed the complicated thermal conditions inside the chip area, and the microstructure was significantly changed as a result. In these studies, dry cutting conditions were always used to reveal the basic deformation mechanisms, as cooling methods could significantly change the microstructure evolution according to Maruda et al. [13,14], which finally influence the cutting conditions, such as the variation in the cutting forces and chip formation. As a result, these phenomena have shown that microstructures play an important role in the mechanical response of materials during the cutting process. 

Several types of constitutive models are developed for a better description of microstructure evolution and its effects on material behaviors during cutting processes. Calamaz et al. [15] raised a modified type of the Johnson–Cook model with an additional term to illustrate the phenomenon of strain-softening, which is attributed to the effects of DRX. This model was then further developed by Sima and Özel [16] for more precise descriptions of the strain softening effects at high strains of Ti6Al4V alloys, which was validated by plenty of experimental data and shows good agreements. However, in their models, the influence of microstructures is only treated as an additional term, which cannot explicitly illustrate its influence on material behaviors. To overcome this shortcoming, Ding et al. [17] reported a dislocation density-based model to describe the constitutive behavior of materials during the cutting process, which could be used to consider the microstructure evolution and update the material behavior explicitly. However, in this model, the temperature effects on the initial stress-state of materials are neglected, which makes the description of the initial yield stress with elevated temperatures inaccurate. Then Denguir et al. [18] introduced a modified JC (Johnson-Cook) model to consider the effects of softening by dynamic recrystallization (DRX), as additional terms and obtained some good results in the orthogonal cutting simulation of OFHC copper, and microstructure evolution could be calculated at the same time by the dislocation-density evolution mechanism. Atmani et al. [19] also presented a mechanical threshold stress (MTS) model with the combination of a dislocation density model to predict the material behaviors and microstructure evolution. However, the constitutive behavior and microstructure evolution were still treated independently in these two models, and the material behaviors were not influenced by the microstructure alteration directly. As a result, it is important to pick a proper model and material to describe its behavior with respect to microstructure evolution during the cutting process and explain the coupled relationship between microstructure and mechanical behavior.

In this study, an orthogonal milling method is conducted to investigate the high-speed machining of OFHC copper with speeds ranging from 750 to 3000 m/min, and SEM and advanced EBSD techniques are used to capture the microstructure information after high-speed cutting processes. OFHC copper is used as the workpiece material due to its simple structure without phase transformation. A dislocation density-based (DDB) constitutive model is used to simulate the cutting process to obtain strain-stress and microstructure information during high-speed cutting processes and illustrate the mechanisms of material behaviors under high-speed cutting conditions. From the experimental and simulation results, it could be concluded that the fluctuation in cutting forces at high cutting speeds is induced by the evolution and distribution of the dislocation density, which is also a key factor influencing the sub-surface formation and fracture during chip separation.

## 2. Simulation and experimentation

### 2.1. Simulation

#### 2.1.1. FEM Model Set-up

**Figure 1:**
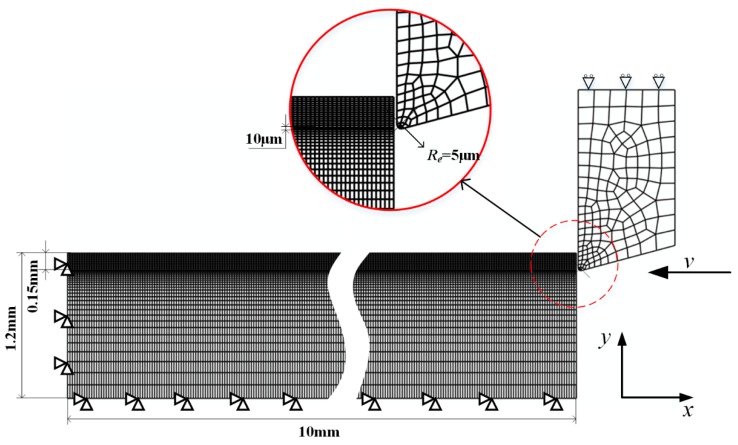
Boundary conditions for 2D orthogonal cutting simulation.

A 2D orthogonal cutting process is modeled by the commercial FEM software Abaqus/Explicit 6.14 with the boundary conditions shown in Figure 1. As cutting is a coupled thermal-mechanical process, thermal-mechanical 2D solid elements (CPE4RT) are applied, and Coulomb’s friction model is used with the friction coefficient 0.4. 

**Figure 2:**
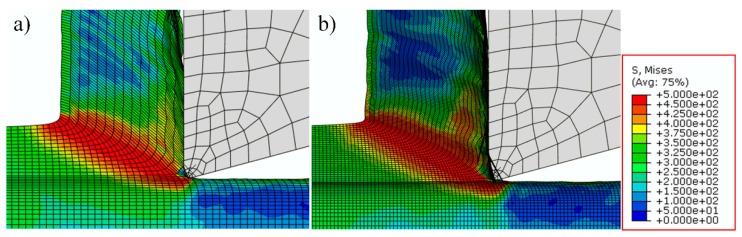
Testing results of mesh sensitivity, (**a**) current mesh and (**b**) finer mesh.

For the chip layer, meshes with dimensions of 20 μm × 10 μm are applied, and the mesh sensitivity has been tested, even if the meshes are refined to a smaller size, the simulation results are less improved while the calculation time increases significantly, and the testing results are shown in Figure 2. 

From this figure, the stress distributions are almost the same between the two different mesh sizes, and the slight difference does not influence the results too much; however, if the mesh sizes of Figure 2b are applied, the calculation time would be about five times more. As a result, meshes shown in Figure 2a are used in this study. Moreover, for the bulk material, finer meshes of about 20 μm × 4 μm are applied only in the area around the machined surface to obtain more precise predictions of the deformation state and microstructure information on the machined surface and save some calculation time.

For the realization of the chip separation, a specified sacrificing layer is used in this model, with the implementation of the Johnson–Cook damage model. From Figure 1, the thickness of this layer is only 10 μm, and the dimension of meshes inside this layer is 20 μm × 5 μm. This model can be expressed as

(1) $$\varepsilon^{f}=(d_{1}+d_{2}e^{d_{3}\sigma^{*}})(1+d_{4}\ln\varepsilon^{*})(1+d_{5}\frac{T-T_{r}}{T_{m}-T_{r}})$$

where σ^*^ is the mean stress normalized by the effective stress, and ε^*^ is the dimensionless plastic strain rate. The parameters *d*_1_, *d*_2_, *d*_3_, *d*_4_, and *d*_5_ are shown in Table 1.

#### 2.1.2. Constitutive Relationship

Estrin et al. [21] firstly presented the DDB (Dislocation-density based) model for 2-D cases with the assumption that material plasticity is induced by the dislocated cells’ movement and annihilation, then Tóth et al. [22] revised the dislocation density evolution law and expanded this model into 3D cases to describe more complicated material behaviors, and finally Ding et al. [17] implemented this model into the machining process to simulate chip formation and grain refinement. The model explicitly calculates the dislocation density evolution and updates the material behavior due to these calculations, which could be expressed as

(2) $${\dot{\rho }}_{c}=\alpha^{*}\frac{1}{\sqrt{3}}\frac{\sqrt{\rho_{w}}}{b}{\dot{\gamma }}_{w}-\beta^{*}\frac{6{\dot{\gamma }}_{c}}{bd{\left(1-f\right)}^{1/3}}-k_{0}{\left(\frac{{\dot{\gamma }}_{c}}{{\dot{\gamma }}_{0}}\right)}^{-1/n}{\dot{\gamma }}_{c}\rho_{c}$$

(3) $${\dot{\rho }}_{w}=\beta^{*}\frac{\sqrt{3}{\dot{\gamma }}_{c}(1-f)\sqrt{\rho_{w}}}{fb}+\beta^{*}\frac{6{\dot{\gamma }}_{c}{\left(1-f\right)}^{2/3}}{bdf}-k_{0}{\left(\frac{{\dot{\gamma }}_{w}}{{\dot{\gamma }}_{0}}\right)}^{-1/n}{\dot{\gamma }}_{w}\rho_{w}$$

(4) $$f=f_{\infty }+(f_{0}-f_{\infty })e^{-\frac{\gamma^{r}}{{\tilde{\gamma }}^{r}}}$$

(5) $$\rho_{tot}=f\rho_{w}+(1-f)\rho_{c}$$

(6) $$d=\frac{K}{\sqrt{\rho_{tot}}}$$

where *ρ_c_* accounts for the dislocation density of the cell interiors, *ρ_w_* accounts for that of the cell walls, α*, β*, and *k*_0_ are the controlling parameters for the evolution rates, *n* is the temperature sensitivity parameter, *f* is the fraction of the dislocation of the cell walls, *b* is the Burgers vector, and *d* is the dislocation cell size. ${\dot{\gamma }}_{c}$ and ${\dot{\gamma }}_{w}$ are the shear strain rates for the cell interiors and cell walls, respectively. *f*_0_ and *f_∞_* are the initial and saturation volume fractions, respectively. The flow stress is updated according to the dislocation density evolution by

(7) $$\tau_{c}^{r}=\alpha Gb\sqrt{\rho_{c}}{\left(\frac{{\dot{\gamma }}_{c}}{{\dot{\gamma }}_{0}}\right)}^{1/m}$$

(8) $$\tau_{w}^{r}=\alpha Gb\sqrt{\rho_{w}}{\left(\frac{{\dot{\gamma }}_{c}}{{\dot{\gamma }}_{0}}\right)}^{1/m}$$

(9) $$\tau^{r}=f\tau_{w}^{r}+(1-f)\tau_{c}^{r}$$

where *G* is the shear modulus and m is a strain-rate sensitivity parameter. Here, the strain rate effects are implemented to modify the calculated values of stress, especially for high strain rates. Moreover, the framework of this constitutive relationship is originated from crystal plasticity, and Steinmetz et al. [23] reported that the stress actually works as a glide resistance for the individual slip system, which should be finally calculated as

(10) $${\hat{\tau }}^{r}=\tau_{0}+\tau^{r}$$

where *τ*_0_ is the solid solution strength which is influenced by the chemical composition, and it is also a temperature-dependent value, and it could be used to compensate the temperature effects on the initial yield stress which is not expressed in Ding’s model. The Taylor factor *M* is applied to transfer the micro-scale calculation to the macro-scale. In addition, as *d* in Equation (5) only accounts for the dislocation cell size or sub-grain size, Derby [24] raised another approach to describe the grain size evolution, which is expressed as

(11) $$\frac{\sigma }{G}{\left(\frac{D_{s}}{b}\right)}^{a}=k$$

where *σ* is the flow stress, *D_s_* is the average grain size at a stable state, *k* and *a* are material constants, and from Andrade et al. [25], *k* is always used as 10 for metals. As cutting is a high strain-rate deformation process, especially for high-speed cutting process, Ding et al. [17] provided controlling parameters suitable for high-speed cutting conditions with high strain rates and Liu et al. [26] also offered the initial resistance stress *τ*_0_ for OFHC copper, as shown in Table 2.

Moreover, according to previous research, the dynamic recovery controlling parameter *k*_0_ is a temperature- and strain rate-dependent parameter, however, in their research, they simplified it as a constant or a linearly increased value with the increase of temperature. In this study, we used a third-order polynomial function to describe it with parameters listed in Table 3, and the equation is expressed as

(12) $$\begin{array}{l} x=\ln\dot{\varepsilon }\\ y=T\\ k_{0}=p_{00}+p_{10}x+p_{01}y+p_{20}x^{2}+p_{11}xy+p_{02}y^{2}+p_{30}x^{3}+p_{21}x^{2}y+p_{12}xy^{2}+p_{03}y^{3} \end{array}$$

**Figure 3:**
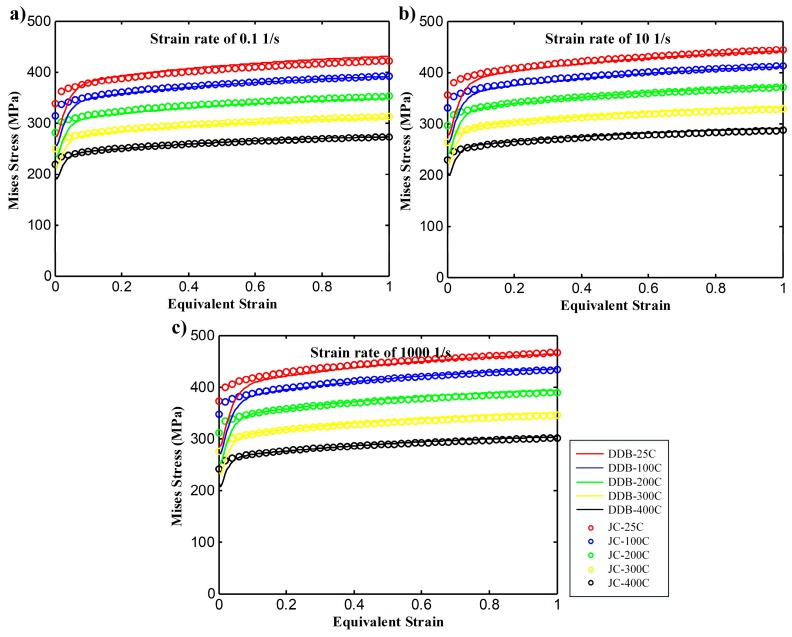
Constitutive behaviors of OFHC copper by DDB model and JC model at different strain rates and temperatures (**a**) strain rate of 0.1 s^−1^, (**b**) strain rate of 10 s^−1^ and (**c**) strain rate of 1000 s^−1^.

For coarse-grained OFHC copper, Meyers et al. [27] raised the parameters for the Johnson–Cook model to describe its constitutive behavior as listed in Table 4, and the DDB model is validated through the comparison with this Johnson–Cook model, as shown in Figure 3. The model is coded as a subroutine VUHARD for the Abaqus/Explicit solver.

### 2.2. Experimentation

**Figure 4:**
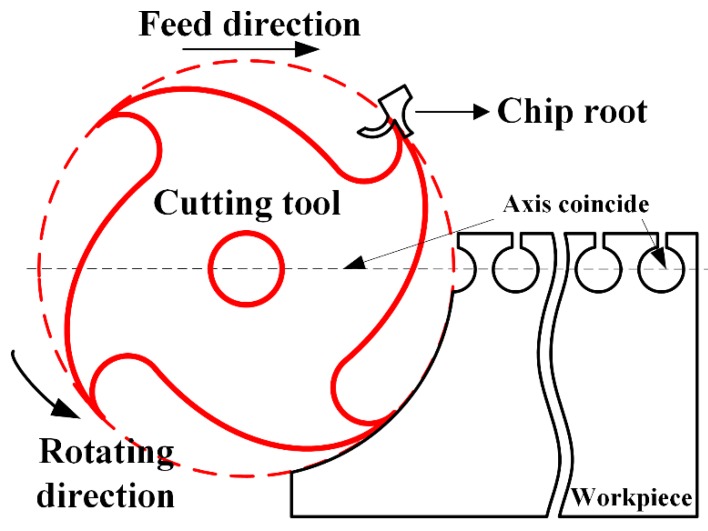
Schematic of Buda’s experimental method.

The cutting experiments were conducted on a five-axis milling machine (model: DMU50, manufactured by DMG MORI, Germany) with the maximum rotation speed of 12,000 RPM. Buda [28] presented a simplified method to obtain chip roots without any auxiliary devices, which is applied to transfer 3D milling processes into 2D orthogonal cutting. The schematic of this experimental method is shown in Figure 4.

**Figure 5:**
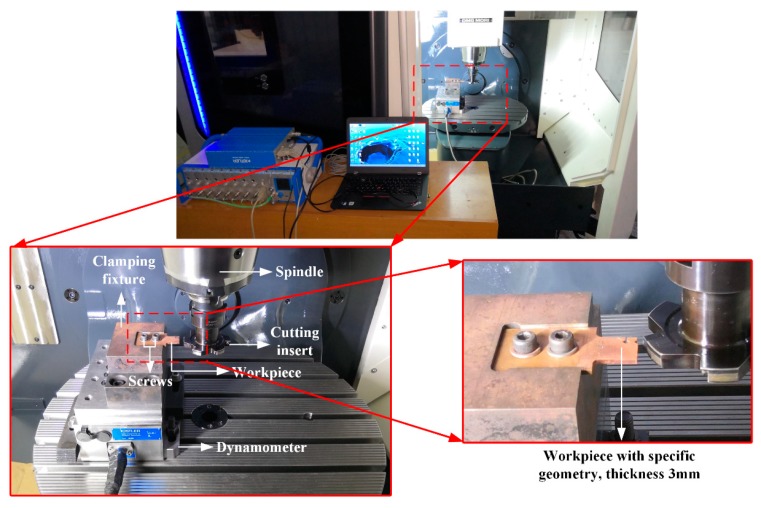
Experimental set-up on the milling machine and workpiece details.

Liu et al. [29] reported a customized cutter with a diameter of 80 mm and specific inclination angle to realize orthogonal cutting, which is adopted in this study as well. The experimental set-up is shown in Figure 5. The uncut chip thickness, which is the same as the feed per tooth during the milling process, is fixed as 0.15 mm in this study, with the cutting speeds increased from 750 m/min to 3000 m/min. The cutting inserts (model: Sandvik N331.1A, manufactured by Sandvik Coromant, Sweden) are uncoated fine-grained carbide ones, with a rake angle of 0° and clearance angle of 15° during the cutting process. The cutting forces are measured by a dynamometer (model: Kistler 9265B, manufactured by Kistler, Switzerland).

To obtain detailed microstructure information, an ion beam slope cutter (model: Leica EM TIC 3X, manufactured by Leica Microsystem, Germany) is used for sample preparation of chips after normal metallurgical treatment, so that high-quality images are obtained through electron backscattered diffraction (EBSD, manufactured by Oxford instruments, England). The EBSD test is conducted by a Scanning Electron Microscope (SEM, model: Hitachi SU3500, manufactured by Hitachi, Japan).

## 3. Results and Discussion

### 3.1. Dislocation Density-Related Microstructure Evolution under High-speed Cutting Conditions

Cutting is a typically severe plastic deformation (SPD) process, which leads to a significant change of microstructures, including refinement and coarsening, depending on both the temperature history and initial grain size. Moreover, Li et al. [5] concluded that the deformation is not uniform during the cutting process, so that the stress and strain states are quite complex inside the cutting zone. As a result, researchers reported it should be a mixed-mechanism for microstructure evolution during the cutting process. However, Roters et al. [30] reviewed the research in this realm and pointed out that no matter what kind of mechanisms it is attributed to, the plastic deformation always relies on the mobility of dislocations, as the dislocation is the carrier of plastic deformation. As a result, for a better understanding of microstructure evolution during cutting, it is very important to investigate how dislocations evolve.

**Figure 6:**
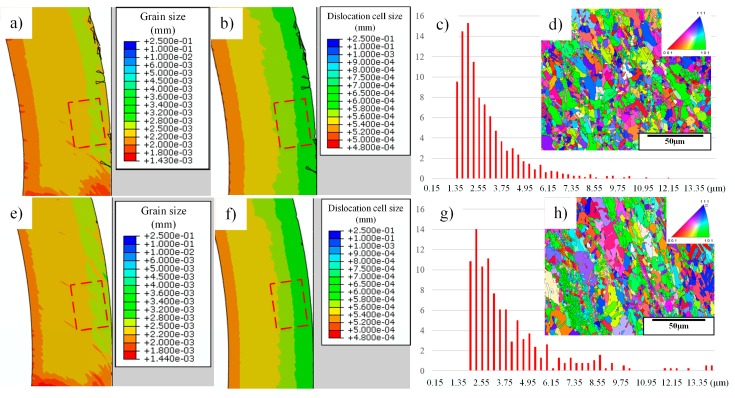
Simulation and experimental results of microstructure information inside the chip area (**a**)–(**d**) are the results for 750 m/min and (**e**)–(**h**) are the results for 1500 m/min. (**a**) and (**e**) are the simulation results of the grain size. (**b**) and (**f**) are the predicted dislocation cell sizes. (**c**) and (**g**) are the experimental data of the actual grain size distribution inside the red frame of (**a**) and (**e**), respectively. (**d**) and (**h**) are the experimental data of the microstructure mapping in the selected area in (**a**) and (**b**), respectively.

With the DDB model, we could obtain the constitutive behavior of material and the information of dislocation densities simultaneously. Moreover, the EBSD technique is used to obtain the information in the selected area of the chips, for the validation of microstructure predictions, which is shown as the red frame inside the chips in Figure 6a,e. As microstructure evolution is induced by dislocation density evolution, the dislocation cell sizes are shown in Figure 6b,f, which could also be referred to as sub-grain sizes. The distribution of grain sizes from the experimental results are shown in Figure 6c,g, and the actual microstructures are shown in Figure 6d,h. 

As the material used in this study has a coarse initial grain size, grain refinement is the dominant phenomena during this high-speed cutting process, which could be easily observed from Figure 6d,h. Moreover, the average grain size is strongly dependent on the flow stress according to Equation (10), while the flow stress is determined by the dislocation density evolution, so the dislocation density evolution is the key factor influencing grain size distribution. From this figure, most grains in the selected area at 750 m/min have a size between 1.5 μm to 2.5 μm, while the value turns to 2 μm to 3 μm at 1500 m/min, which means the average grain size increases slightly with the increase of cutting speeds. For the predicted values, it lies in the same area as the experimental data, however, the discrepancies are quite small, which are 2.2 μm to 2.8 μm at 750 m/min and 2.5 μm to 3.2 μm at 1500 m/min, respectively. 

Moreover, from Figure 6b,f, the related dislocation cell size, or sub-grain size in the measured area is also smaller at 750 m/min, which is consistent with the results of the grain sizes. During this process, grain refinement could be explained by DRX, and continuous dynamic recrystallization (cDRX) should be the dominant mechanism as most of the refined grains are the results of sub-grain rotation inside initial coarse grains, but not nucleates along the initial grain boundaries, as shown in Figure 6d,h. This phenomenon is quite different from that of the lower speed cutting OFHC copper, according to Guo et al. [2], as discontinuous dynamic recrystallization (dDRX) or mixed mechanisms of dDRX and cDRX are the dominant characteristics due to the large strain and low strain rate at conventional cutting conditions, while the percentage of cDRX increases significantly with the increase of cutting speeds. As a result, we can deduce that higher cutting speeds induce a higher growth rate of dislocation cells and sub-grains, and these cells and sub-grains finally form the refined grains.

### 3.2. Dislocation Density Evolution-Induced Fluctuation of Cutting Forces

**Figure 7:**
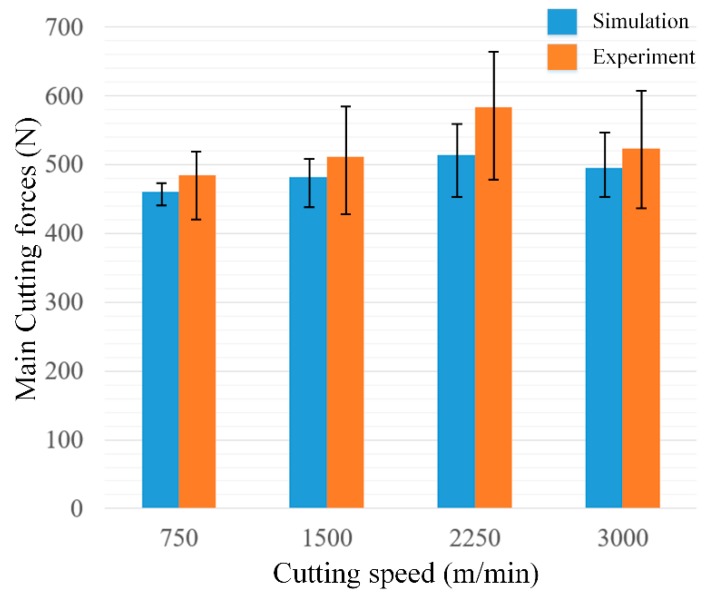
Simulation and experimental results of the main cutting forces.

Cutting forces are important factors showing the mechanical process during machining, and the fluctuations of simulation and experimental results are shown in Figure 7, respectively. Maximum and minimum values are used to show the fluctuation of cutting forces during high-speed cutting, and the fluctuation of experimental data is larger than that of simulation results, as milling is adopted in experiments for the realization of high cutting speeds. The average cutting forces of simulation and experimental results are in good agreement. Moreover, from Figure 7, the main cutting forces hit a threshold when cutting speeds become higher than 2250 m/min, and although there is just a slight decrease at 3000 m/min, it shows a trend that the cutting forces become lower if the cutting speed continues to increase.

Cutting forces are significantly influenced by the material properties, which is determined by microstructures. Moreover, according to Hall–Petch relationship, materials could be strengthened due to the grain refinement; however, the change in grain size is not so significant according to the simulation and experimental results shown in Figure 6, so the variation in the cutting forces between 750 m/min and 1500 m/min is very slight, as shown in Figure 7. 

**Figure 8:**
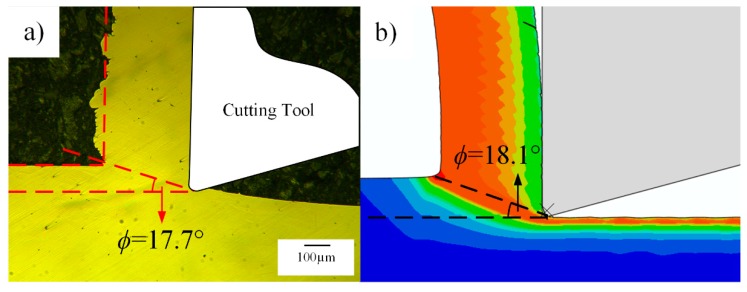
Shear angle measured from (**a**) experimental results and (**b**) simulation results.

The cutting forces are influenced by the change of chip morphologies, as no serrated chips are formed during machining of OFHC copper, shear angle is the key factor to characterize the chip morphology. Through Buda’s method shown in Figure 4, chip roots can be easily obtained in experiments, using the measurements of shear angles, as shown in Figure 8.

The shear angles for all the cutting conditions are listed in Table 5, from which a good agreement between the simulation and experimental results is shown.

The fluctuation of cutting forces increases with the increase of cutting speeds according to the experimental results from Figure 7. On the experimental side, this fluctuation may be caused by tool vibration and change of chip morphologies, which has been investigated in a previous study; Xu et al. [31] reported a similar phenomenon in the machining of titanium alloys, and they concluded the reasons for the change of chip morphology was due to the formation of serrated chips. Liu et al. [32] also presented experimental work on the machining of the Ti6Al4V alloy and concluded that the cutting forces fluctuated as the fracture mechanism transformed during serrated chips formation with the increase of cutting speeds. However, in this study, these factors are neglected in the simulation work, so the fluctuation of simulation results should be induced by some inner parameters of materials. Moreover, during the machining of OFHC copper, continuous chips are always obtained even though the cutting speed is very high, so the internal variables of the materials would be the key factors, which is attributed to dislocation density in this study. 

**Figure 9:**
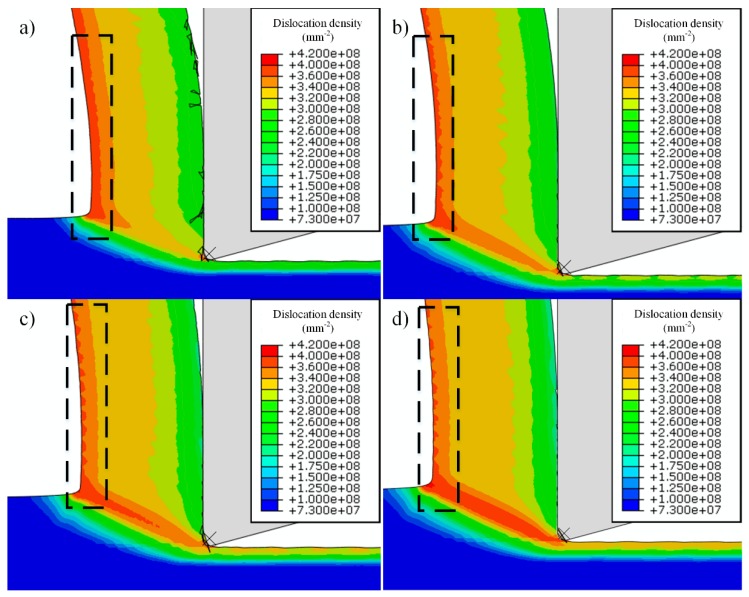
The distribution of the dislocation density at different cutting speeds, (**a**) 750 m/min, (**b**) 1500 m/min, (**c**) 2250 m/min and (**d**) 3000 m/min, area in the black rectangle show the distribution of the dislocation density in free surface of chips.

From the simulation, with the increase of cutting speeds, the distribution of dislocation densities inside the chips shows a significant difference, especially for the area of the free surface. At lower cutting speeds of 750 m/min, high-density dislocations distribute continuously in this area, then it shows some fluctuations at 1500 m/min, and finally it turns to discontinuous when cutting speeds exceed 2250 m/min, as shown in Figure 9.

**Figure 10:**
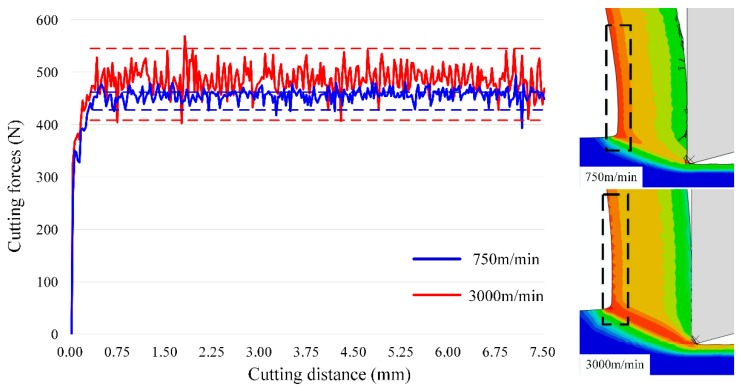
Fluctuation of cutting forces induced by the dislocation density evolution at different cutting speeds.

Although chip morphology is continuous during machining of OFHC copper within 750 m/min to 3000 m/min, this phenomenon of dislocation density distribution shows a similar trend as the cutting forces fluctuate. The fluctuation is also quite weak at 750 m/min, and is more severe at 2250 m/min and 3000 m/min, at the same time the discontinuous distribution of dislocation density appears, as shown in Figure 10.

For the fluctuation of cutting forces, it could be induced by tool wear or tool vibration in the experimentation, while for the simulation side, these factors are neglected and these fluctuations still exist, which means it is influenced by the material behavior. In this figure, the cutting conditions at 750 m/min and 3000 m/min are used due to the significant change of dislocation density distribution shown in Figure 9, respectively. From Figure 10, the fluctuation is very small at 750 m/min, while it is enlarged to a high value when cutting speed is elevated to 3000 m/min. This wave-shaped distribution or fluctuation of dislocation density only appears at high-speed cutting conditions due to the high strain and strain rate, while for low-speed cutting OFHC copper, the continuous distribution is always observed [17]. Moreover, Ding and Shin [33] reported that the increase of microhardness depends on dislocation density during plastic deformation as well, which can be expressed as

(13) $$\Delta h=k_{h}M_{t}\alpha_{0}Gb\sqrt{\rho_{tot}}$$

where *k_h_*, *M_t_* and *α*_0_ are all material constants, which means the hardness is controlled by the dislocation density. From Figure 9, the average dislocation density inside the chip area increases with the increasing cutting speeds, which finally leads to the strengthening of the material and the increase of cutting forces. As a result, we deduce that the change of dislocation density distribution is one of the key factors influencing the variation in the cutting forces, especially for high-cutting speeds.

**Figure 11:**
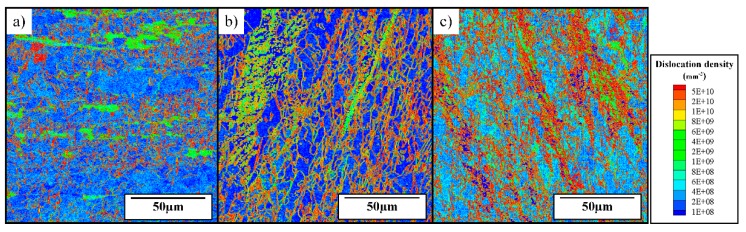
Distribution of GNDs (geometrically necessary dislocations) inside chips with the cutting speeds of (**a**) 750 m/min, (**b**) 1500 m/min and (**c**) 2250 m/min.

Moreover, in the dislocation-density based model described as Equations (1)–(9), only statistically stored dislocations (SSDs) are considered and a Taylor factor is used for homogenization. However, geometrically necessary dislocations (GNDs) also have a significant influence on fluctuations in cutting forces, as they are induced by strain gradient and material heterogeneity. Although GNDs are not taken into consideration in the original model assumptions, they can be measured through experiments, which relies on the Euler angles information measured through the advanced EBSD technique, as Tóth and Gu [34] raised the method to transfer Euler angles into GNDs, and the distribution of GNDs inside chips is shown in Figure 11.

From Figure 11, the GND density shows a much higher value than the simulation results of SSD (statistically stored dislocation) density. Jorge–Badiola et al. [35] presents a method to estimate the total dislocation density of both GNDs and SSDs, which can be expressed as

(14) $$\rho_{S+G}=\rho_{S}+\rho_{G}={\left(\frac{\sigma }{\alpha Gb}\right)}^{2}$$

According to this equation, we can use the flow stress to estimate the average value of GNDs, which lies in the same level as shown in Figure 11. Moreover, in Figure 11, just a few high-density GNDs appear along grain boundaries at 750 m/min, while when cutting speeds increase to 1500 m/min and 2250 m/min, high-density GNDs at grain boundaries increase significantly, and fluctuations in cutting forces reach the peak value at the same time, which means GND is also one of the key factors influencing the variation in the cutting force, which plays a more important role at higher cutting speeds due to materials heterogeneity, these factors are not reported to be significant at conventional cutting conditions [2,17]. As a result, the cutting forces fluctuation at high cutting speeds is believed to be influenced by the evolution of both SSDs and GNDs, and the material behaviors are more sensitive to the heterogeneity at high cutting speeds, which makes GNDs more important. Although GND induced by strain gradient is not considered in the current model, it will be implemented in the future work.

### 3.3. Effects of the Dislocation Density Evolution on Surface Formation and Chip Separation

**Figure 12:**
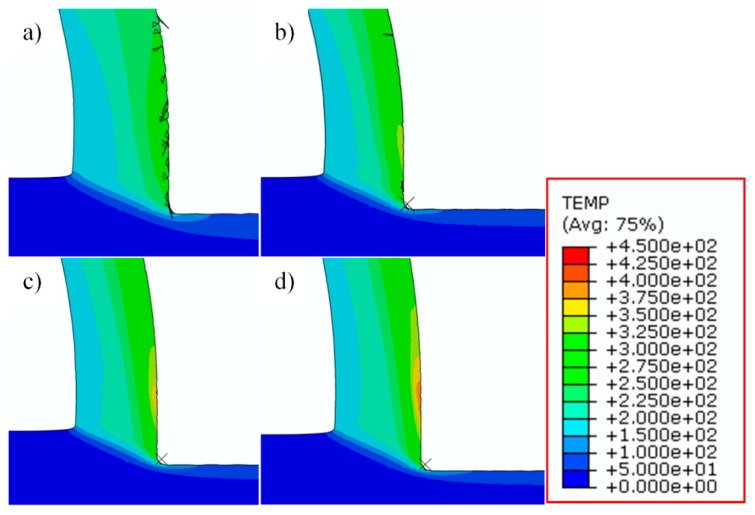
Temperature distributions inside chips at different cutting speeds, (**a**) 750 m/min, (**b**) 1500 m/min, (**c**) 2250 m/min and (**d**) 3000 m/min.

The evolution of dislocation density in the machined surface is related to its evolution inside chips as well. From Figure 9, with the increase of cutting speeds, higher dislocation densities appear in the primary shear zone, which also induces the increase of dislocation densities in machined surfaces. Moreover, the machined surface with high-density dislocations can be divided into two layers, the top layer has a depth of about 5 μm, while the thickness of the whole layer is about 12 μm. When cutting speeds increase to 1500 m/min, dislocation densities within the depth of 5 μm become lower than that at the depth ranging from 5 μm to 12 μm, and the phenomenon is more obvious at higher cutting speeds. In this case, the annihilation of dislocations inside chips increases with the increasing cutting speeds due to the increase of temperature, especially in the tool-chip interface, which leads to the decrease of dislocation densities in this area. However, in primary shear zones and machined surfaces, temperatures do not increase so much as in tool-chip interface, and the plastic deformation becomes more severe as cutting speeds increase, which has enhanced the activation process of dislocations. The distributions of temperatures are shown in Figure 12.

**Figure 13:**
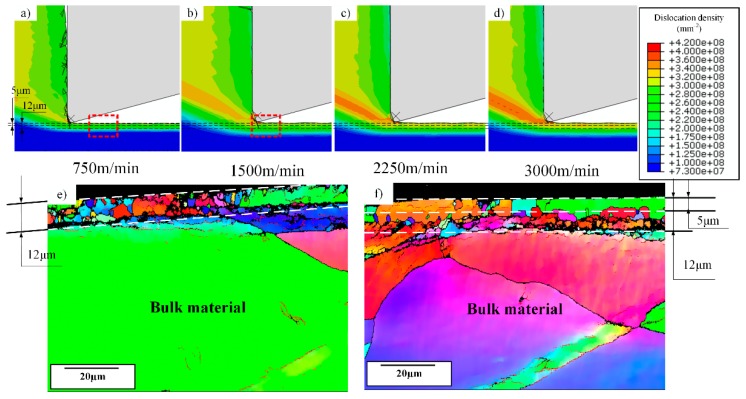
Distribution of statistically stored dislocation densities in sub-surface with the increase of cutting speeds, (**a**)–(**d**) are the simulation results from 750 m/min to 3000 m/min, (**e**) and (**f**) are the experimental data inside the red rectangular in (**a**) and (**b**), respectively.

From this figure, temperature is not the dominant factor influencing the dislocation density evolution inside primary shear zone, while the temperature at tool-chip interface increases significantly with the increase of cutting speeds. Meanwhile, for machined surfaces, the same phenomenon of two layers is also observed from the experimental data of grain sizes in the machined surface by EBSD, as shown in Figure 13. A sub-surface layer appears at 1500 m/min with finer grains, and the refined area at 750 m/min and 1500 m/min both have a depth of about 12 μm, which is consistent with the simulation results.

Moreover, a periodical distribution of dislocation densities appears in the machined surface with the increase of cutting speeds, which is similar to the fluctuation of dislocation densities inside chips as shown in Figure 9. Xu et al. [36] reported that similar periodical distribution of surface contour in the machining of Ti6Al4V, which is attributed to serrated chips formation as they have the same form of fluctuation. However, in this study, chips are continuous and the morphology of machined surface is not changed, the only parameter that has been changed is the dislocation density, and this phenomenon is obvious when cutting speeds exceed 1500 m/min, which means it is a strain- and strain rate-dependent phenomenon.

**Figure 14:**
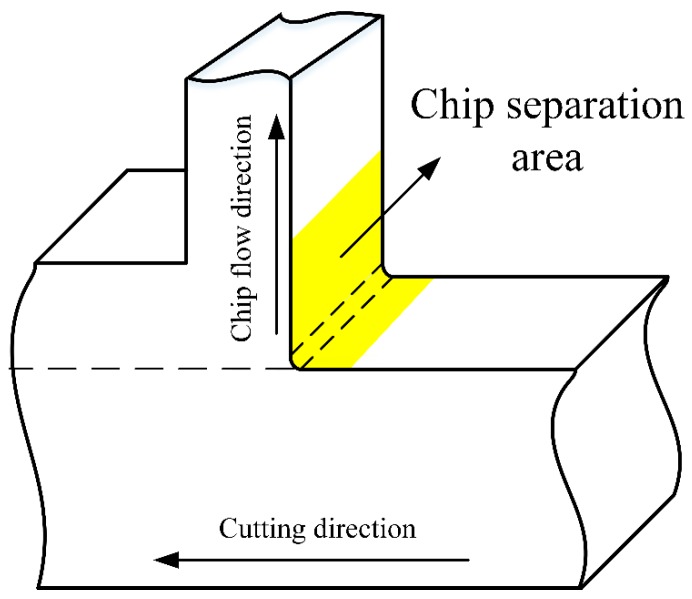
Schematic of chip root sample and chip separation area.

Moreover, the fracture happens during the surface formation process, and fracture characteristics show a variation during this process as well. Chip root samples are used to investigate fracture behavior at chip separation area, which are obtained through experiments as illustrated in Section 2.1, and the schematic of the observation zone is shown in Figure 14.

**Figure 15:**
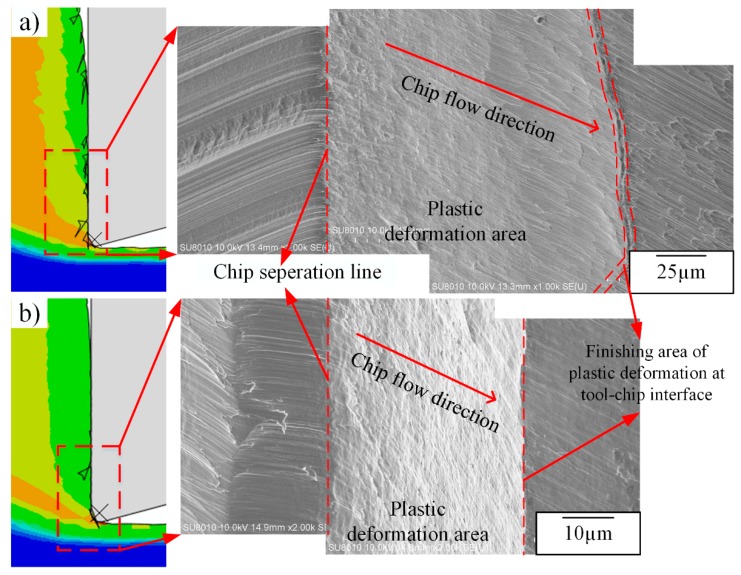
Fracture characteristics during chip separation at different cutting speeds, (**a**) 750 m/min and (**b**) 1500 m/min.

Dislocation density is higher at the tool-chip interface due to the lower temperature at lower cutting speeds, which is shown from Figure 9 and Figure 13; however, burrs are also formed in the same area. When cutting speeds increase, dislocation density at the tool-chip interface decreases as the temperature becomes higher, and the amount of burrs decreases simultaneously. Hashimura et al. [37] conducted that burrs formation is attributed to the effects of edge radius during the interaction between cutting tools and workpieces, which also record the characteristics of plastic deformation and crack propagation during the chip separation process. Moreover, Ko and Dornfeld [38] pointed out that burr size reduces with the increase of cutting speeds, which is similar to the simulation and experimental results shown in this study. At 750 m/min, many elements remain undeleted at the tool-chip interface, and at the same time, more burrs are formed with a larger deformation zone during chip separation as well. While for 1500 m/min, most elements of the sacrificing layer are deleted during the fracture process and few remain undeleted, in this case fewer burrs are obtained with a smaller deformation zone as well, which can be observed from the chip root samples, as shown in Figure 14. Moreover, the undeleted elements of the sacrificing layer can be regarded as burrs, which appear more frequently when plastic deformation zone is formed more sufficiently, as shown in Figure 15. Moreover, from Figure 9, the value of dislocation density in the fracture area around the tool-tip is lower when more burrs are formed at low cutting speeds.

In this study, as the fracture behavior of the material is not investigated quantitatively, a qualitative summary is deduced based on the comparison between the simulation and experimental results. The dislocation density is a key factor influencing the plastic deformation during the cutting process, and its distribution and evolution finally lead to the sub-surface layer formation in the machined surface and burr formation at tool-chip interface.

## 4. Conclusions

In this study, dislocation density induced mechanical behavior of OFHC copper during high-speed cutting is investigated through numerical and experimental methods. DDB model is used to describe the constitutive behaviors in order to reveal the relationship between dislocation density evolution and mechanical response of materials during the cutting process. The results are discussed in three different aspects, which are microstructure evolution, cutting forces variation and fracture characteristics, respectively, and the conclusion can be summarized as follows:

- Dislocation is the carrier of plastic deformation in the cutting process, and the fluctuation of the cutting forces is induced by its evolution and distribution. Both SSDs and GNDs are introduced to describe this phenomenon. The distribution of SSDs is simulated by the DDB model, which has shown a discontinuous distribution inside the chips when the cutting forces begin to fluctuate at high cutting speeds. The GNDs are used for supplementary illustration of the dislocation effects, which are obtained through the EBSD technique, which is also a key factor for the variation of the cutting forces with respect to the cutting speeds, and the values increase with the increase of cutting speeds, especially at grain boundaries due to the heterogeneity of materials.
- The periodical distribution of the dislocation densities appears in both the chips and machined surfaces with the increase of the cutting speeds. This layer in the machined surface has a certain thickness of about 12 μm, and the value of the dislocation densities decreases in the chips while an increase in machined surfaces is seen as the cutting speeds become higher, which is also related to the fluctuation of the cutting forces.
- With the increase of cutting speeds, the dislocation densities around the tool-chip interface decrease, meanwhile, the area of the plastic deformation during the chip separation decreases with fewer burrs formed.

## Figures and Tables

**Table 1:** Johnson–Cook (JC) damage model parameters for OFHC copper [20].

Parameter	*d*_1_	*d*_2_	*d*_3_	*d*_4_	*d*_5_
Value	0.54	4.89	−3.03	0.014	1.12

**Table 2:** DDB model parameters for coarse-grained OFHC copper [17,26].

Parameter	Value	Parameter	Value
*τ*_0_	30 MPa	*K*	10
α*	0.04	*d*_0_	200 μm
β*	0.01	$\dot{\gamma_{0}}$	4000 s^−1^
*f*_0_	0.25	*G*	48 GPa
*f_∞_*	0.165	*A*	30,000
*ρ*_*w*,0_	1 × 10^7^ mm^−2^	*B*	14,900
*ρ*_*c*,0_	1 × 10^8^ mm^−2^	*M*	3.06
*b*	0.256 nm		

**Table 3:** Controlling parameters of the polynomial function for *k*_0_.

Parameter	Value	Parameter	Value
*p*_00_	9.72	*p*_02_	−1.701 × 10^−5^
*p*_10_	0.2124	*p*_30_	4.266 × 10^−4^
*p*_01_	0.006083	*p*_21_	9.068 × 10^−5^
*p*_20_	−0.01218	*p*_12_	2.898 × 10^−6^
*p*_11_	−3.54 × 10^−4^	*p*_03_	3.255 × 10^−8^

**Table 4:** Johnson-Cook parameters for coarse-grained OFHC copper [27].

Material	*A* (MPa)	*B* (MPa)	*C*	*n*	*m*	*T_r_* (°C)	*T_m_* (°C)	έ (1/s)
OFHC	320	80	0.012	0.324	1	25	1083	0.001

**Table 5:** Comparison of shear angles and chip thickness.

Cutting Speed (m/min)	Experimental Results		Simulation (°)	
	Shear Angle (°)	Thickness (mm)	Shear Angle (°)	Thickness (mm)
750	12.8	0.62–0.68	14.4	0.58
1500	14.5	0.55–0.58	16.7	0.5
2250	17.7	0.45–0.48	18.4	0.45
3000	18.3	0.44–0.46	19.0	0.44
